# Phonological Assessment Instrument: evidence of reliability

**DOI:** 10.1590/2317-1782/20232022303en

**Published:** 2023-11-10

**Authors:** Giovana Sopezack Alves, Camila Botura, Ana Carolina Sartori Bernardi, Letícia Pacheco Ribas

**Affiliations:** 1 Curso de Fonoaudiologia, Universidade Federal de Ciências da Saúde de Porto Alegre - UFCSPA - Porto Alegre (RS), Brasil.; 2 Departamento de Fonoaudiologia, Universidade Federal de Ciências da Saúde de Porto Alegre - UFCSPA - Porto Alegre (RS), Brasil.

**Keywords:** Validation Studies, Speech, Speech Articulation Tests, Assessment, Child, Language Tests, Phonological Disorder, Validity of the Tests, Speech Therapy

## Abstract

**Purpose:**

To present evidence of intra- and inter-rater reliability and internal consistency of the Phonological Assessment Instrument scores, so that it can be considered reliable and valid for use in clinical practice.

**Methods:**

179 audio recordings of the instrument's speech samples were analyzed. The collection was carried out from its application in the period of 5 months in children aged from five to eight years and 11 months. Three expert judges transcribed the speech production of each child into the software, which generated performance reports. The speech data of each child were compared between these evaluators, who were trained and experienced in phonetic transcription, to verify the agreement of the instrument scores. For the reliability analysis, the internal consistency was verified using Cronbach's Alpha and the intra and inter-rater reliability using the Intraclass Correlation Coefficient.

**Results:**

The Phonological Assessment Instrument showed evidence of high internal consistency, with scores indicating excellent reliability for the assessment of Brazilian Portuguese phonemes, as well as adequate agreement among the judges regarding the instrument scores.

**Conclusion:**

The instrument presented robust evidence of reliability, being a reliable and safe option to be used in Brazilian research and clinical practice to evaluate the phonological system of Brazilian children.

## INTRODUCTION

The typical phonological development occurs from a pattern in the order of phonemes domain, which needs to be stabilized until approximately five years and six months of age in all syllabic positions^(1)^. Consonants can occupy different positions in Brazilian Portuguese (BP), namely: Simple Onset, which marks the consonant that fills the beginning of the syllable and can be initial (ISO) when it is at the beginning of the word, or medial (MSO) when it is in the middle of the word; Complex Onset, also being classified as initial (ICO) or medial (MCO), which indicates the junction of a lateral or non-lateral liquid (/l/ or /r/) with another consonant at the beginning of the syllable. it should be noted that there are restrictions on the consonants that can occupy the referred position, as for ICO in conjunction with /l/, only the phonemes /p/, /b/, /k/, /g/, and /f/ can appear in the first position, while in conjunction with /r/, it would be all plosive phonemes and /f/; In MCO, in conjunction with /l/, only the phonemes /p/, /b can appear /, /t/, /k/, /g/ and /f/, and in conjunction with /r/, would be all plosive phonemes, and labial fricatives; in Coda, which marks the consonant that is at the end of the syllable, which can be in the middle of the word indicated as medial (MC) or final (FC) when it is at the end of the last syllable (only the nasal, fricative, lateral consonants and non-lateral may occupy this position, being underspecified in relation to the point of articulation and/or sonority and depending on the linguistic context and/or the speaking community)^(1,2)^. To verify whether or not a given phoneme was acquired, it is necessary to analyze the correct production frequency of sounds in each syllabic position since the same phoneme can occupy different positions and structures. They may have indicators of 75% or more to demonstrate that the phoneme is acquired in that particular syllabic context and 75% to 50% for the phoneme that is in the acquisition process, and below 50% for the non-acquired phoneme^(2)^.

When there is no complete acquisition of the phonological system within the expected period, the child presents omissions and/or substitutions of phonemes, mainly in consonants, which may cause speech unintelligibility^(3)^. This condition, called Phonological Disorder (PD), is idiopathic and characterized by a set of signs, namely: having persistent difficulty in speech production; age over four years; present consonant changes or omissions in speech; having auditory thresholds within normal limits; having normal intellectual abilities; no changes in lexicon and syntax concerning expressive language; absence of neurological alterations or evident organic causes and having the ability to understand speech^(4,5,)^. Thus, it is essential to confirm these criteria and carry out an effective phonological assessment, since only this will describe in detail the changes in the child's speech and in what phase of acquisition each phoneme is located. It should be noted that in this study the expression Phonological Disorder is used considering the same as ASHA (American Speech-Language-Hearing Association), which considers it as a Speech Sound Disorder, and that the DSM-5^(4)^ refers to as Speech Disorder. However, the term Phonological Disorder was chosen because both in ASHA and DSM-5^(4)^, it is exposed in a general way, encompassing both articulation and phonological disorders in the same denomination, and this study specifically refers to PD.

The prevalence of PD in Brazil varies^(6-10)^, with the highest value found being approximately 25% of the child population aged between six and 12 years^(11)^. This fact generates a lot of demand for speech-language pathology evaluation and treatment. There are some tests to help in the evaluation and diagnosis. Among those available and most used in Brazil^(12)^: the Child's Phonological Assessment (AFC)^(13)^ and the child language test ABFW-Children's Language Test-Phonology^(14)^. Such instruments demand considerable time for application and analysis, requiring training and prior specific knowledge of the applicator so that a correct assessment can be made, in addition to requiring detailed technical care, since the analysis is done manually. In addition, these instruments do not have psychometric indicators of validity and reliability, which can impair the safety of clinical evidence to draw an accurate diagnosis, adequate conduct, and correct intervention planning^(15)^. There are other speech assessment instruments, but little publicized and/or not available for use, such as the Phonological Assessment Instrument (INFONO)^(12)^ and the Speech Assessment Instrument for Acoustic Analysis (IAFAC)^(16)^.

A test is valid when it measures what it purports to measure. In this sense, it needs to go through validation steps. This process includes construct validity, which refers to the direct way of verifying the hypothesis of the legitimacy of the behavioral representation of the items; content validity, which consists of verifying whether the test constitutes a representative sample of a finite universe of behaviors; and response-process validity, which concerns the degree of effectiveness that the test has in predicting a specific performance of a subject^(17)^.

In addition, an instrument needs to bring evidence of reliability for it to be considered reliable^(18)^. Therefore, the same test needs to be stable and consistent to reproduce an equivalent result from different examiners or from the same evaluator at different times. In this sense, there are three ways to verify reliability: through internal consistency, inter-rater reliability, and intra-rater reliability. The first seeks to verify whether all subparts of the instrument measure the same characteristic, that is, whether the responses to the test are consistent. The second involves the independent participation of two or more evaluators who will complete the instrument, and later it is verified if there was equivalence in the score obtained between them. On the other hand, intra-evaluator reliability indicates that the same judge should fill in the instrument data and perform the analysis at two different times, independently of the first application, to verify whether the result remains stable and consistent over time^(18,19)^.

Due to the lack of specific criteria in the usual phonological assessment instruments in Brazil, Prof. Dr. Letícia Pacheco Ribas created the Phonological Assessment Instrument (IAF) in 2007, which aims to quantify and qualify what the child is presenting in their phonological system. The IAF is a computerized instrument, made up of 123 words with an image corresponding to each lexical item, which arose from the need to evaluate speech based on a practical, quick-to-apply protocol that could analyze children's phonological system more carefully and efficiently. Given the above, this study aimed to present evidence of intra- and inter-rater reliability, and internal consistency, of the Phonological Assessment Instrument scores, so that it can be considered reliable and valid for use in clinical practice.

## METHOD

This research corresponds to an observational, cross-sectional, controlled, descriptive, and quantitative study, whose data were used to verify the evidence of the reliability of the IAF. The study was approved by the Research Ethics Committee of a federal university under number 5.045.533.

### Sample

After carrying out the content validity stages with the expert judges and the response-process validity^^a^^ , the study of the necessary sample size was carried out. It was calculated to be 25%, according to the maximum estimate of the prevalence of the diagnosis of PD for the child population^(11)^. To determine a Kappa coefficient of 0.80, which indicates good agreement, and for a significance of 5% and power of 80%, the result was data from at least 165 children for a representative sample.

To carry out the reliability analysis, the audios of the IAF speech recordings were used, previously collected for a research study on the validity of the response-process, applied to a group of children aged between five years and eight years and 11 months. This collection process occurred in the period from August to December, from a public school in Porto Alegre, selected from a database with 219 evaluations. Forty children who had evident organic causes, hearing, neurological, and/or cognitive alterations, school difficulties, history of neuro psychomotor delay, and/or intercurrences during pregnancy or childbirth were excluded. These exclusions we based on the analysis of the anamnesis responses and the preliminary assessments carried out in the previous study that verified the validity of response-process.

Information from participants with characteristics of typical phonological development or with PD was included, totaling 179 children for the study. The information was checked through a previous assessment and reports in interviews with those responsible. All parents or guardians signed the Free and Informed Consent Term and the Authorization for the use of Audio, and in the case of children over seven years old, they also signed the Term of Assent.

### Instrument

IAF^^b^^ is a software designed to evaluate the child's speech sound system efficiently, thoroughly, and optimally. The instrument consists of 123 words belonging to children's vocabulary, extracted from popular children's stories, easily represented in an image or photo, and are of the noun type, with an image corresponding to each lexical item. The items were carefully selected so that they included all consonant phonemes in all syllabic positions in Brazilian Portuguese (BP), with five occurrences of each phoneme and syllabic position, totaling 235 phonemic possibilities. The collection of the child's speech should occur by naming each of the images, by observing the illustrations or photographs, which takes approximately ten minutes for the application. The evaluator must record the audio of the speech collection, and later, listen and observe the children's elicitations and register the information to the software. This process takes around 15 to 30 minutes, depending on the evaluator's practice and skill. After entering the data into the instrument, the results are automatically generated and expressed in descriptive and qualitative reports by the degree of speech severity, contrastive analysis, phonological processes, and change in distinctive features.

### Procedures

The analysis of internal consistency and inter-rater reliability was performed based on the judgment of three expert judges with training and experience in phonetic transcription. They were presented with the audio of the speech collections of the 179 children, blinded, and asked to mark the phonetic correspondence of each target in the software. Based on the transcript, the instrument generated all reports. The data of each child were compared between the judges to verify the degree of agreement of the IAF scores.

For the intra-evaluator reliability analysis, the same evaluators analyzed again the same audio samples from the speech collections of the original application. This occurred independently from the first analysis and the results were compared individually. That is, it was verified whether the results of the first and second analyses were equivalent between the same evaluator. This stage was carried out with the audio of 18 children (approximately 10% of the sample), randomly drawn, and evaluated by the same evaluators three months after the first analysis. Research and validation studies suggest performing the second analysis between seven and 14 days after the first assessment^(18,19)^ in order not to have a change in the child's development. In this study, it was decided to carry out the analysis over a longer period, since it is done from audio already recorded and, therefore, would not have alteration in the individual's phonological system. In addition, a longer listening time for the audio recordings could decrease the possibility of remembering phonological exchanges performed by the children, with the reassessment being independent and without bias from the first analysis made by the evaluators.

The data used in the inter and intra-rater reliability for the analysis of the degree of severity were obtained from the Percentage of Correct Consonants - Revised^(20)^. As for the contrastive analysis, in both analyses, the percentage of correct answers for each phoneme was verified for each child. For the internal consistency analysis, the data were generated from the cross-judgment of the evaluators, who classified the phonemes into three categories for the application of the statistical calculation (acquired phoneme, in acquisition or not acquired), and analyzed the frequency of correct production of the sounds for each child. The phonological processes were organized as having or not having the presence of each process.

For this study, reports on the degree of speech severity, contrastive analysis, and phonological processes were used. The results of the distinctive features were not presented, since such data are contemplated in the report of the phonological processes, as they are the same object analyzed by different theoretical perspectives, and that result in the same findings.

### Data analysis

The results of the reliability analysis stage were approached quantitatively. Analyses were performed using SPSS software, version 28 for Windows.

#### Internal consistency

To verify the internal consistency, Cronbach's Alpha coefficient was calculated, which is the most used measure to assess reliability^(18)^. It measures the degree of covariance between the items of a scale and allows analysis of the consistency of the instrument, calculating the correlation that exists between each test item and the rest of the items or the total of the items^(17)^. Values range from 0 to 1, with values greater than 0.7 considered ideal, suggesting adequate reliability^(18)^. For values greater than 0.90, it is assumed that there is redundancy or duplication, indicating that several items are measuring the same element of the construct, requiring the elimination of these redundant items^(21)^.

#### Inter-rater reliability

To compare the average of the total number of phonological processes and to evaluate the judges' compliance with the results of the contrastive analysis and the degree of speech severity, the Intraclass Correlation Coefficient (ICC) was performed. It is suitable for measuring the correlation of ratings between two or more raters when there is a quantitative variable. Values range from 0 to 1. The closer to 1, the greater the agreement between raters. If less than 0.5, the agreement is weak. Between 0.5-0.75, it is moderate. Between 0.75-0.9, the agreement is good. If greater than 0.9, the agreement is excellent^(22).^

#### Intra-rater reliability

To assess the agreement of the judges, that is, to measure the degree of conformity of the assessments between two different moments, in the results of the degree of speech severity and the contrastive analysis, the ICC was used. The values adopted as a criterion are the same as those referred to for inter-rater reliability.

## RESULTS

Evidence of reliability was verified from the results collected by the IAF. To verify the instrument's internal consistency, the results were analyzed using Cronbach's Alpha coefficient for each phoneme in each syllabic position in Brazilian Portuguese (BP). The instrument had a coefficient of 0.88, indicating high internal consistency (above 0.7), showing excellent reliability of the scores that assess BP phonemes.

Table 1 shows the average of the total number of processes found by each evaluator, through the ICC. The agreement between the evaluators, regarding the number of processes, had averages ranging from 2.37 to 3.2 per child, with an amplitude of 0.65, and ICC indicating good agreement. This indicates consonance between the evaluators concerning the phonological processes found in the participants.

**Table 1:** Average of the total amount of phonological processes among judges

Judge						ICC	CI 95%
**A**		B		C			
M	SD	M	SD	M	SD		
**2.50**	**1.99**	**3.02**	**2.06**	**2.37**	**1.85**	**0.89**	**0.84 0.92**

Caption: M = Mean; SD = Standard Deviation; ICC = Intraclass Correlation Coefficient; CI = Confidence Interval

**Figure 1:**
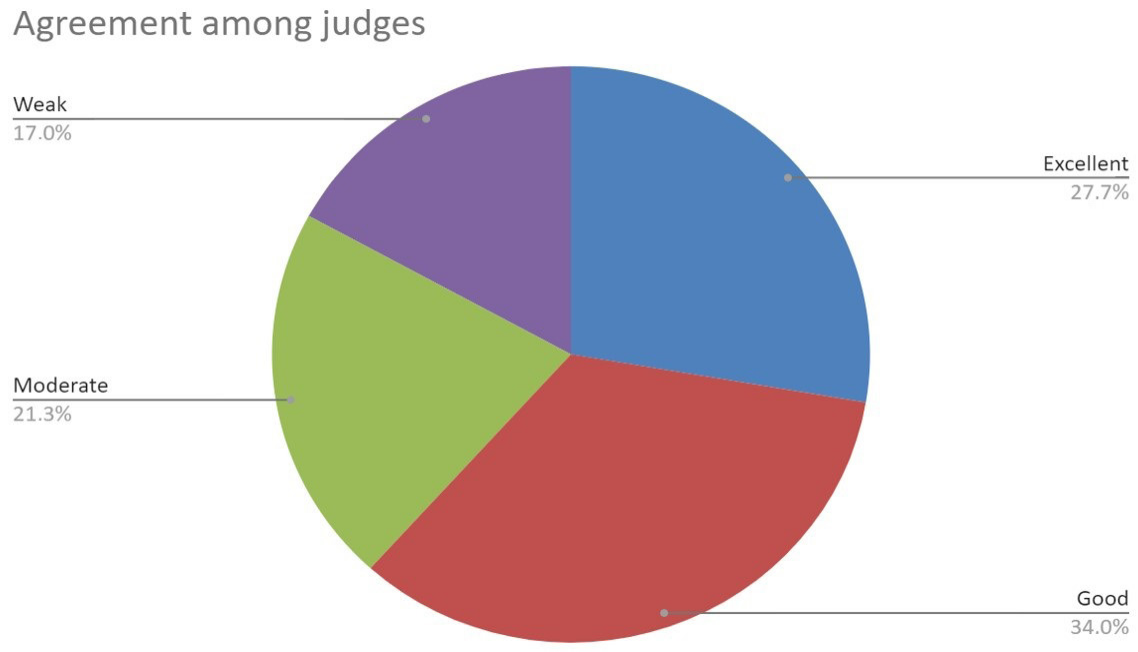
Ratio of inter-rater agreement on the correct production of phonemes in each syllabic position in BP

As for the evidence of inter-rater reliability of the contrastive analysis, Table 2 shows the reliability between the judges, measured by the agreement of the correct production of the phonemes in each syllabic position of BP, through the ICC. The agreement had values above 0.90 for /ʃ/ in ISO and MSO; /ʒ/ in ISO and MSO; the /R/ in ISO; the /ʎ/ in MSO; the /r/ in MSO, ICO, MCO, and MC; the /l/ in ICO and MCO; and the /s/ in MC. The agreement obtained values between 0.75 and 0.90, being considered good for most other phonemes, as shown in Figure 1.

**Table 2:** Inter-rater agreement measured by the correct production of phonemes in each syllabic position in BP using the ICC

Phonemes	Positions					
	ISO	MSO	ICO	MCO	MC	FC
/p/	0.42*	0.81	-	-	-	-
/b/	0.72	0.72	-	-	-	-
/t/	0.71	0.71	-	-	-	-
/d/	0.83	0.84	-	-	-	-
/k/	0.86	0.90	-	-	-	-
/g/	0.88	0.78	-	-	-	-
/f/	0.32*	-0.01*	-	-	-	-
/v/	0.73	0.63	-	-	-	-
/s/	0.71	0.89	-	-	0.91	0.47
/z/	0.71	0.86	-	-	-	-
/ʃ/	0.91	0.95	-	-	-	-
/ʒ/	0.93	0.92	-	-	-	-
/m/	-0.01*	0.36*	-	-	-	-
/n/	0.51*	0.82	-	-	0.76	0.41
/ɲ/	-	0.61	-	-	-	-
/R/	0.96	0.87	-	-	-	-
/l/	0.83	0.82	0.97	0.93	0.77	0.24*
/ʎ/	-	0.91	-	-	-	-
/r/	-	0.94	0.96	0.95	0.91	0.90

* Sample is highly homogeneous

Caption: ISO = Initial Simple Onset; MSO = Medial Simple Onset; ICO = Initial Complex Onset; MCO = Medial Complex Onset; MC = Medial Coda; FC = Final Coda

There was moderate agreement only on /b/ in ISO and MSO; /t/ in ISO and MSO; /v/ in ISO and MSO; /s/ in ISO; /z/ in ISO; /n/ in ISO; and /ɲ/ in MSO. In most of the phonemes in which agreement was considered weak, there was an indication of a highly homogeneous sample.

Table 3 presents the inter-rater and intra-rater comparison regarding the result of the speech severity degree of each child, which was measured by the ICC. The result showed that the ICC was above 0.90 in all occurrences, indicating excellent agreement both between the evaluators and between each evaluator at two different moments.

**Table 3:** Inter-rater and intra-rater reliability measured by the degree of speech severity

Inter-rater	ICC	CI 95%
	0.97	0.96-0.98
**Intra-rater**	**ICC**	**CI 95%**
Judge A	0.98	0.95-0.99
Judge B	0.98	0.91-0.99
Judge C	0.98	0.94-0.99

Caption: ICC = Intraclass Correlation Coefficient; CI = Confidence Interval

Regarding the intra-rater reliability of the contrastive analysis, Table 4 shows the reliability of each judge. This was measured by the agreement of the correct production of phonemes in each syllabic position of BP, through the ICC. Only the phonemes in which it was possible to carry out the statistical analysis were listed, as in some there was no variability of standard deviation. The ICC only performs the analysis if there is such variability. When there is no such variability, that is, total agreement, it is not possible to determine the statistical coefficient due to the characteristics of the ICC analysis.

**Table 4:** Intra-rater reliability measured by the correct production of phonemes in each syllabic position in BP, using the ICC

Phonemes	Judge A	CI 95%	Judge B	CI 95%	Judge C	CI 95%
ISO /g/	0.92	0.80 - 0.97	0.69	0.16 - 0.88	0.96	0.89 - 0.98
ISO /v/	*	*	-0.25	-2.75 - 0.54	*	*
ISO /z/	0.89	0.72 - 0.99	0.76	0.37 - 0.91	0.37	-0.66 - 0.76
ISO /ʃ/	0.98	0.95 - 0.99	0.99	0.98 - 0.99	0.98	0.96 - 0.99
ISO /n/	*	*	0.36	-0.57 - 0.75	-0.15	-2.01 - 0.56
MSO /ʒ/	0.95	0.87 - 0.98	0.98	0.96 - 0.99	0.99	0.98 - 0.99
MSO /ɲ/	*	*	0.79	0.45 - 0.92	*	*
MSO /ʎ/	0.84	0.58 - 0.94	*	*	0.62	-0.00 - 0.85
MSO /r/	0.98	0.95 - 0.99	0.99	0.99 - 0.99	0.97	0.93 - 0.99
ICO /l/	0.98	0.97 - 0.99	0.99	0.98 - 0.99	0.98	0.95 - 0.99
ICO /r/	0.96	0.91 - 0.98	0.99	0.97 - 0.99	0.96	0.88 - 0.98
MCO /l/	0.97	0.92 - 0.99	0.99	0.97 - 0.99	0.96	0.91 - 0.98
MCO /r/	0.98	0.97 - 0.99	0.98	0.97 - 0.99	0.96	0.90 - 0.98
MC /s/	*	*	*	*	0.87	0.67 - 0.95
MC /l/	0.86	0.64 - 0.95	0.95	0.86 - 0.98	0.58	-0.14 - 0.84
MC /r/	0.80	0.48 - 0.92	0.98	0.96 - 0.99	0.81	0.49 - 0.93
FC /n/	*	*	0.76	0.40 - 0.91	*	*
FC /r/	0.92	0.76 - 0.97	0.93	0.83 - 0.97	0.86	0.63 - 0.94

* It was not possible to perform the ICC due to a lack of variability in the standard deviations

Caption: ISO = Initial Simple Onset; MSO = Medial Simple Onset; ICO = Initial Complex Onset; MCO = Medial Complex Onset; MC = Medial Coda; FC = Final Coda; CI = Confidence Interval

Agreement between evaluators was considered excellent, with values above 0.9 for most phonemes. There were several phonemes with agreement classified as good, with values between 0.75 and 0.90, indicating adequate reliability among the judges. Evaluator A had agreement considered excellent or good in all listed phonemes. Evaluator B had the phoneme /g/ in ISO considered moderate, with values between 0.50 to 0.75, in addition to having the phonemes /v/ in ISO and /n/ in ISO classified as weak, with lower values to 0.50. Evaluator C had agreement considered moderate for the phonemes /ʎ/ in MSO and /l/ in MC, and agreement considered weak for the phoneme /z/ in ISO.

## DISCUSSION

There is a recognized relevance in the use of speech evaluation instruments for speech therapy clinical practice^(23)^. However, to be considered reliable, it is necessary to go through precise measurement steps, namely: validity and reliability^(15-19)^. Given the objective of this study and its results, the IAF proved to be a pertinent and appropriate instrument to analyze the phonological system of BP-speaking children in detail, in addition to determining the degree of PD severity, having adequate indicators, and satisfactory reliability.

Regarding the evidence of the reliability of the IAF, the internal consistency proved to be excellent, since it was above the indicator considered minimally ideal (0.70), indicating a strong reliability. This result shows the absence of redundancy or duplication in the instrument's items. Other national speech-language assessment instruments also used Cronbach's Alpha to analyze internal consistency: the Phonological Assessment Instrument (INFONO)^(12)^, which assesses phonology; and the speech discrimination task with pseudowords^(24)^, created to assess the auditory discrimination ability of speech sounds. Both obtained results above 0.70, having indicators considered appropriate. In a protocol constructed to assess the oral language comprehension of children aged between two and six years old^(25)^, the internal consistency ranged from 0.60 to 0.70, classified as moderate. As for a study on the internal consistency of the task of evaluating syntactic competenc^ies (26)^, Cronbach's Alpha ranged from 0.00 to 0.47, being insufficient to validate the reliability of the instrument.

In a systematic review study^(27)^, which analyzed the validation procedures used by speech therapy instruments for the assessment of oral language, published in national journals, it was evident that only five instruments, out of a total of 21, carried out reliability analyses based on internal consistency. Such results demonstrate the scarcity of validated instruments in relation to reliability, preventing the accuracy of precise data in the evaluation of speech therapy clinical practice.

In the study mentioned^(27)^, the sample calculation for defining the sample size was presented in only one study out of the 21 included in the systematic review, proving a significant limitation in the validation studies of speech tests. The IAF, on the other hand, demonstrates both the internal consistency indicators and the sample calculation and can be considered a reliable instrument for use in clinical practice and research.

The instruments need to go through stages of validity and reliability to measure what they are intended to measure and for the results to represent the analyzed construct, so as not to compromise the accuracy of the assessment and diagnosis^(12)^. Thus, an instrument created to evaluate the phonological system needs to analyze all the phonemes in all the syllable positions of its target language, in order to make a correct judgment about the presence or absence of a diagnosis of PD. This structure is advocated in the IAF to enable a thorough analysis of the child's phonological profile and is rarely found in other language assessment tests. Following this same parameter, only the Fuzzy Linguistic Model^(28)^, designed to classify the severity of PD, and the aforementioned INFONO^(12)^ were found. Thus, an instrument created to evaluate the phonological system needs to analyze all the phonemes in all the syllable positions of its target language, in order to make a correct judgment about the presence or absence of a diagnosis of PD.

Identifying the types of phonological processes and the substitutions that the child performs is information that collaborates with the phonological assessment^(26,29)^, but for an instrument to be able to add this analysis, it needs to be able to assess all the phonemic possibilities in all the syllable positions in a quali-quantitative manner^(28)^. The IAF has this attribute, providing detailed information on the phonemes and all their possibilities of occurrence, being an adequate instrument to include the observation of the phonological processes.

Regarding the means of inter-rater agreement for phonological processes, the results indicated adequate reliability for this analysis. However, it should be noted that despite being complementary data to outline the choice of therapy model, the analysis of phonological processes is neither essential nor necessary^(2)^. It does not explain, in a detailed and judicious way, the organization of the phonological system and its functioning in relation to each phoneme in each syllable position of BP, because only in this way is it feasible and appropriate to outline the therapeutic approach^(29)^. Therefore, the analysis of the use of phonological processes is additional data for the evaluation, but not fundamental, since the verification of the contrastive analysis provides the guiding and primordial aspects to know the functioning of the phonological system of an individual^(29)^.

In addition to internal consistency, inter-rater and intra-rater reliability were performed to test the reliability of the instrument. An international study designed to assess speech production in Turkish children^(30)^ also followed the same steps to verify the evidence of reliability, as well as another national research study^(12)^. In both steps, the IAF indicators presented in the current study for the analysis of the degree of severity were considered excellent, demonstrating that it is reliable data for its use in evaluations and diagnoses in clinical practice.

The two steps for the contrastive analysis of the IAF were also carried out, in which it was observed that most of the phonemes presented reliability between excellent and good. However, both stages had some phonemes with moderate and weak indicators (Table 2). Regarding inter-rater reliability, this occurrence may have been affected by the fact that the sample was highly homogeneous, compromising the statistical analysis and making it difficult to discriminate the production of phonemes among sample participants. The homogeneity of the sample can affect the ICC. Therefore, if the research population is very homogeneous, that is, when they are very similar according to a certain characteristic (resulting in a low standard deviation), there is greater difficulty in discriminating between individuals and the ICC deviates from 1 (close to 0). As an example, the phoneme /p/ in ISO was indicative of a highly homogeneous sample, possibly due to the lack of variability in the subjects' responses (data for this phoneme in this position were practically identical for all children), and thus, there was a low standard deviation, making it difficult to perform the statistical calculation of ICC, deviating from 1. The variation found in Table 2 is due to the varied amplitude of ICC found among the evaluators.

Furthermore, it should be mentioned that the reliability of an instrument can be influenced by the experience and individual criteria of each evaluator^(12)^. Disagreements in the inter-rater reliability of the contrastive analysis may have occurred due to the hearing ability and the raters' parameters. This can be verified in the intra-rater reliability, since rater B and C, even having most of the phonemes with excellent reliability, had some phonemes with moderate and weak indicators, while rater A had only good and excellent indicators (Table 4). Likewise, another study^(28)^ also had different indicators among the judges, which affected the agreement indicators in view of reliability. However, it should be mentioned that the high ICC values for the inter- and intra-rater reliability of the IAF in most phonemes indicate that there is excellent agreement between the judges (Table 2). Thus, the IAF proves to be reliable in classifying the acquisition phase^(1)^ in which each child is, in each syllable position, facilitating the evaluation and diagnosis, as well as the therapeutic planning and individual evolution.

The BP phonology assessment instruments most applied and disseminated currently in the country^(12)^ are the AFC^(13)^ and the ABFW^(14)^, as previously mentioned, which are very important tools for the diagnostic process of Brazilian speech therapists. However, despite such importance, they still lack psychometric studies of validity and reliability, which could affect the safety of using the measures of these instruments. Thus, this study is relevant for the advancement of speech therapy, since the IAF becomes a valid and qualified option to be used in evaluations of children with suspected PD and/or with alterations in the phonological system, in addition to being an instrument that enables a quick and thorough analysis, since the software quickly produces a performance report.

The IAF was designed to contribute to clinical practice, helping in the assessment of children's phonology, and presented excellent reliability results. Despite this, this study has some limitations, such as: being structured for BP speakers in general, but the data being exclusive to speakers of only one sample from a public school in Rio Grande do Sul, which may be an obstacle to generalization; not having used standardized indexes to calibrate the hearing evaluation, allowing the evaluators' criteria; and not presenting reliability coefficients for each age group, gender and type of school, since the variability of the sample is composed only of students from public education.

The present study demonstrated that the IAF is a reliable instrument for use and applicability, providing evidence of the reliability of the scores, however, other studies should be carried out in search of other evidence of validity, such as the test consequences stage, standardization, standardization, and item analysis. It should be noted that the IAF software was developed to assess any age group, however, it was validated for children aged over five years, respecting the stabilization period of phonological acquisition and the set of signs that characterize the PD, in addition to age Sample.

## CONCLUSION

The IAF presented robust evidence of reliability, demonstrating good internal consistency scores, which points to excellent reliability of the instrument's items, in addition to inter and intra-rater reliability. Thus, there was an adequate agreement between the results provided by the IAF, both when applied by different evaluators and when applied individually at different times. Because it is a study that followed psychometric validation parameters, the evidence presented demonstrates the quality of the instrument and strengthens the safety of using its measures. It is concluded that the IAF is a reliable and safe option to be used in Brazilian research and in clinical practice to assess children's phonological systems.

## Footnotes

a Research being developed by Ana Carolina Sartori Bernardi, in 2022, at the University of Health Sciences in Porto Alegre.

b Psychometric studies on the instrument's properties of content validity, response-process, and construct validity are in production.
