# Supplementary material for: Phonological Assessment Instrument: evidence of reliability
Source: Codas. 2023 Nov 10;36(1):e20220303. doi: 10.1590/2317-1782/20232022303en (PMC10715575; doi:10.1590/2317-1782/20232022303en)
Supplement: Translation

# Translation: Instrumento de Avaliação Fonológica: evidências de fidedignidade

**DOI:** 10.1590/2317-1782/20232022303pt

**Authors:** Giovana Sopezack Alves, Camila Botura, Ana Carolina Sartori Bernardi, Letícia Pacheco Ribas

**Affiliations:** 1 Curso de Fonoaudiologia, Universidade Federal de Ciências da Saúde de Porto Alegre - UFCSPA - Porto Alegre (RS), Brasil.; 2 Departamento de Fonoaudiologia, Universidade Federal de Ciências da Saúde de Porto Alegre - UFCSPA - Porto Alegre (RS), Brasil.

**Keywords:** Estudos de Validação, Testes de Articulação da Fala, Avaliação, Criança, Testes de Linguagem, Transtorno Fonológico, Validade dos Testes, Fonoaudiologia

## Abstract

**Objetivo:**

Apresentar evidências de fidedignidade intra e interavaliadores, e de consistência interna, dos escores do Instrumento de Avaliação Fonológica, a fim de que possa ser considerado fidedigno e válido para a utilização na prática clínica.

**Método:**

Foram analisados 179 áudios dos registros de fala do instrumento, cuja coleta foi realizada a partir da sua aplicação no período de 5 meses em crianças na faixa etária dos cinco aos oito anos e 11 meses. Três juízes especialistas transcreveram no *software* a produção de fala de cada criança, o qual gerou relatórios de desempenho. Os dados de fala de cada criança foram comparados entre esses avaliadores, treinados e experientes em transcrição fonética, para verificar a concordância dos escores do instrumento. Para a análise da fidedignidade, foi verificada a consistência interna pelo *Alpha de Cronbach* e a confiabilidade intra e interavaliadores por meio do Coeficiente de Correlação Intraclasse.

**Resultados:**

O Instrumento de Avaliação Fonológica apresentou evidências de alta consistência interna, com escores indicando excelente fidedignidade para avaliação dos fonemas do Português Brasileiro, como também uma adequada concordância entre os juízes acerca dos escores do instrumento.

**Conclusão:**

O instrumento apresentou evidências robustas de fidedignidade, sendo uma opção confiável e segura para ser utilizado em pesquisas brasileiras e na prática clínica para avaliar o sistema fonológico de crianças.

## INTRODUÇÃO

O desenvolvimento fonológico típico ocorre a partir de um padrão na ordem de domínio dos fonemas, que precisa estar estabilizado até aproximadamente cinco anos e seis meses de idade em todas as posições silábicas^(1)^. As consoantes podem ocupar diferentes posições no Português Brasileiro (PB), sendo: Onset Simples, que marca a consoante que preenche o início da sílaba, podendo ser inicial (OSI) quando está no início da palavra, ou medial (OSM), quando está no meio da palavra; Onset Complexo, também sendo classificada em inicial (OCI) ou medial (OCM), que indica a junção de uma líquida lateral ou não lateral (/l/ ou /r/) com outra consoante no início da sílaba (há de se atentar que há restrições quanto às consoantes que podem ocupar a posição referida, sendo que para OCI em junção com /l/, apenas os fonemas /p/, /b/, /k/, /g/ e /f/ podem figurar na primeira posição, enquanto que em junção com o /r/, seriam todos os fonemas plosivos e o /f/; Já em OCM em junção com o /l/, podem figurar apenas os fonemas /p/, /b/, /t/, /k/, /g/ e /f/, e em junção com /r/, seriam todos os fonemas plosivos, e as fricativas labiais); em Coda, a qual marca a consoante que está no final da sílaba, que pode ser no meio da palavra apontada como medial (CM) ou final (CF) quando estiver no final da última sílaba (apenas as consoantes nasal, fricativa, lateral e não lateral podem ocupar essa posição, sendo subespecificadas em relação à ponto de articulação e/ou sonoridade e dependente do contexto linguístico e/ou da comunidade falante)^(1,2)^. Para verificar se um determinado fonema foi ou não adquirido, necessita-se analisar a frequência de produção correta dos sons em cada posição silábica (visto que um mesmo fonema pode ocupar diferentes posições e estruturas), tendo indicadores de 75% ou mais para demonstrar que o fonema está adquirido naquele determinado contexto silábico, 75% a 50% para o fonema que está em processo de aquisição, e abaixo de 50% para fonema não adquirido^(2)^.

Quando não há aquisição integral do sistema fonológico no período esperado, a criança apresenta omissões e/ou substituições de fonemas, principalmente nas consoantes, podendo ocasionar ininteligibilidade de fala^(3)^. Tal condição, denominada Transtorno Fonológico (TF), é idiopática e caracterizada por um conjunto de sinais, sendo eles: ter dificuldade persistente para produção de fala; idade superior a quatro anos; apresentar trocas ou omissões consonantais na fala; ter limiares auditivos dentro dos padrões de normalidade; ter habilidades intelectuais normais; sem alterações no léxico e na sintaxe com relação à linguagem expressiva; ausência de alterações neurológicas ou causas orgânicas evidentes e ter capacidade de compreensão da fala^(4,5)^. Dessa forma, faz-se indispensável a confirmação desses critérios e a realização de uma efetiva avaliação fonológica, já que somente esta descreverá detalhadamente as trocas na fala da criança e em que fase de aquisição cada fonema se encontra. Cabe salientar que neste trabalho se utiliza a expressão Transtorno Fonológico considerando o mesmo que a ASHA (American Speech-Language-Hearing Association), considera como Transtorno dos Sons da Fala e que o DSM-5^(4)^ refere de Transtorno da Fala. Porém, opta-se por Transtorno Fonológico, pois tanto na ASHA quanto no DSM-5^(4)^, ele é exposto de forma geral, englobando tanto os transtornos de articulação quanto fonológico na mesma denominação, e refere-se neste estudo especificamente o TF.

A prevalência de TF no Brasil é variada^(6-10)^, sendo que o valor mais alto encontrado foi de aproximadamente 25% da população infantil entre seis a 12 anos^(11)^. Esse fato gera muita demanda pela avaliação e tratamento fonoaudiológico, e por conta disso, existem alguns testes para auxiliar na avaliação e diagnóstico, sendo, entre os disponíveis, conhecidos e mais utilizados no Brasil^(12)^: a Avaliação Fonológica da Criança (AFC)^(13)^ e o teste de linguagem infantil ABFW-Teste de Linguagem Infantil-Fonologia^(14)^. Tais instrumentos demandam um considerável tempo para aplicação e análise, necessitando de treinamento e conhecimento específico prévio do aplicador para que seja feita uma avaliação correta, além de precisar de um cuidado técnico minucioso, visto que a análise é feita manualmente. Além disso, os referidos instrumentos não possuem indicadores psicométricos de validade e de fidedignidade, o que pode prejudicar a segurança das evidências clínicas para traçar um diagnóstico preciso, uma conduta adequada e um planejamento de intervenção correto^(15)^. Existem outros instrumentos de avaliação de fala, mas pouco divulgados e/ou não disponíveis para uso, como o Instrumento de Avaliação Fonológica (INFONO)^(12)^ e o Instrumento de Avaliação de Fala para Análise Acústica (IAFAC)^(16)^.

Um teste é válido quando, de fato, mede o que se propõe a medir. Nesse sentido, necessita passar por etapas de validação. Esse processo abrange: a validade de construto, que se refere à maneira direta de verificar a hipótese da legitimidade da representação comportamental dos itens; a validade de conteúdo, que consiste em verificar se o teste constitui uma amostra representativa de um universo finito de comportamentos; e a validade de processo-resposta, que diz respeito ao grau de eficácia que o teste tem em predizer um desempenho específico de um sujeito^(17)^.

Além disso, um instrumento precisa trazer evidências de fidedignidade para que seja considerado confiável^(18)^. Sendo assim, um mesmo teste precisa ser estável e consistente para reproduzir um resultado equivalente a partir de examinadores diferentes ou de um mesmo avaliador em momentos distintos. Nesse sentido, há três maneiras de verificar a fidedignidade: pela consistência interna, pela confiabilidade interavaliadores e pela confiabilidade intra-avaliador. A primeira busca verificar se todas as subpartes do instrumento medem a mesma característica, ou seja, se as respostas ao teste são consistentes. Já a segunda envolve a participação independente de dois ou mais avaliadores, os quais preencherão o instrumento e, posteriormente, é verificado se houve equivalência na pontuação obtida entre eles. Por outro lado, a confiabilidade intra-avaliador indica que o mesmo juiz deverá preencher os dados do instrumento e realizar a análise em dois momentos distintos, de forma independente da primeira aplicação, para verificar se o resultado permanece estável e consistente ao longo do tempo^(18,19)^.

Devido à falta de critérios específicos nos instrumentos usuais de avaliação fonológica no Brasil, foi elaborado em 2007 por Profª Drª Letícia Pacheco Ribas o Instrumento de Avaliação Fonológica (IAF), que visa quantificar e qualificar o que a criança está apresentando em seu sistema fonológico. O IAF é um instrumento computadorizado, formado por 123 palavras com uma imagem correspondendo cada item lexical, que surgiu pela necessidade de avaliar a fala a partir de um protocolo prático, de rápida aplicação e que pudesse analisar de forma mais criteriosa e eficiente o sistema fonológico infantil. Tendo em vista o supracitado, este estudo teve o objetivo de apresentar evidências de fidedignidade intra e interavaliadores, e de consistência interna, dos escores do Instrumento de Avaliação Fonológica, a fim de que possa ser considerado fidedigno e válido para a utilização na prática clínica.

## MÉTODO

Esta pesquisa corresponde a um estudo observacional, transversal controlado, descritivo e quantitativo, cujos dados foram utilizados para verificar a evidência de fidedignidade do IAF. O estudo foi aprovado pelo Comitê de Ética em Pesquisa (CEP) de uma universidade federal sob parecer número 5.045.533.

### Amostra

Após serem realizadas as etapas de validade de conteúdo com os juízes especialistas e a validade de processo-resposta^^a^^ , foi realizado o estudo do tamanho amostral necessário, calculado com 25%, conforme estimativa máxima de prevalência do diagnóstico de TF para a população infantil^(11)^. Para determinar um coeficiente Kappa de 0,80, que indica boa concordância, e para significância de 5% e poder de 80%, o resultado foi de, minimamente, dados de 165 crianças para uma amostra representativa.

Para realizar a análise da fidedignidade, foram utilizados os áudios dos registros de fala do IAF, previamente coletados para uma pesquisa sobre a validade de processo-resposta, aplicados em um grupo de crianças, entre cinco anos e oito anos e 11 meses, no período de agosto a dezembro, de uma escola pública de Porto Alegre, selecionadas de um banco de dados com 219 avaliações. Foram excluídas 40 crianças que possuíam causas orgânicas evidentes, alterações auditivas, neurológicas e/ou cognitivas, dificuldades escolares, histórico de atraso neuropsicomotor e/ou de intercorrências em gravidez ou parto, a partir da análise das respostas da anamnese e das avaliações realizadas preliminarmente no estudo anterior que verificou a validade de processo-resposta.

Foram incluídas as informações dos participantes com características de desenvolvimento fonológico típico ou com TF, totalizando 179 crianças para o estudo. As informações foram conferidas por meio de avaliação prévia e relatos em entrevista com os responsáveis. Todos os pais ou responsáveis assinaram o Termo de Consentimento Livre e Esclarecido e a Autorização para uso de Áudio, e no caso de crianças acima de sete anos, essas também assinaram o Termo de Assentimento.

### Instrumento

O IAF^^b^^ é um *software* delineado para avaliar o sistema de sons da fala infantil de forma eficiente, minuciosa e otimizada. O instrumento é formado por 123 palavras pertencentes ao vocabulário infantil, extraídas de histórias infantis populares, facilmente representadas em imagem ou foto e são do tipo substantivo, com uma imagem correspondente a cada item lexical. Os itens foram criteriosamente selecionados de forma com que contemplassem todos os fonemas consonantais em todas as posições silábicas do PB, com cinco ocorrências de cada fonema e posição silábica, totalizando 235 possibilidades fonêmicas. A coleta da fala da criança deve ocorrer a partir da nomeação de cada uma das imagens, pela observação das ilustrações ou fotografias, que leva aproximadamente dez minutos para a aplicação. O avaliador deve gravar o áudio da coleta de fala, e posteriormente, ouvir e observar as elicitações das crianças e registrar as informações ao *software*. Tal processo leva em torno de 15 a 30 minutos, dependendo da prática e habilidade do avaliador. Após a inserção dos dados no instrumento, os resultados são gerados automaticamente e expressos em relatórios descritivos e qualitativos por grau de severidade de fala, análise contrastiva, processos fonológicos e mudança de traços distintivos.

### Procedimentos

A análise da consistência interna e da confiabilidade interavaliadores foi realizada a partir do julgamento de três juízes especialistas com treinamento e experiência em transcrição fonética. A estes, foram apresentados os áudios das coletas de fala das 179 crianças, de forma cega, e solicitado que fizessem a marcação no *software* da correspondência fonética de cada alvo. Com base na transcrição, o instrumento gerou todos os relatórios. Os dados de cada criança foram comparados entre os juízes para verificar o grau de concordância dos escores do IAF.

Para a análise da confiabilidade intra-avaliadores, os mesmos avaliadores analisaram novamente os mesmos áudios das amostras das coletas de fala da aplicação original, de forma independente da primeira análise, e os resultados foram comparados de forma individual, ou seja, conferiu-se se os resultados da primeira e da segunda análise foram equivalentes entre o mesmo avaliador. Esta etapa foi realizada com áudios de 18 crianças (aproximadamente 10% da amostra), sorteados aleatoriamente e avaliados pelos mesmos avaliadores três meses após a primeira análise. Pesquisas e estudos sobre validação sugerem realizar a segunda análise entre sete a 14 dias da primeira avaliação^(18,19)^ para não ter mudança no desenvolvimento da criança. Neste estudo, optou-se por realizar a análise em um tempo superior, visto que ela é feita a partir de um áudio já gravado e, dessa forma, não teria alteração no sistema fonológico do indivíduo. Além disso, um tempo maior de oitiva dos áudios poderia diminuir a possibilidade de lembrança de trocas fonológicas realizadas pelas crianças, sendo a reavaliação totalmente independente e sem viés da primeira análise feita pelos avaliadores.

Os dados utilizados na confiabilidade inter e intra-avaliador para as análises do grau de severidade foram obtidos a partir do Percentual de Consoantes Corretas- Revisado^(20)^. Já para a análise contrastiva, em ambas as análises, foi verificada a porcentagem de acertos de cada fonema, para cada criança. Para a análise da consistência interna, os dados foram gerados a partir do julgamento cruzado dos avaliadores, os quais classificaram os fonemas em três categorias para a aplicação do cálculo estatístico (fonema adquirido, em aquisição ou não adquirido), e analisaram a frequência de produção correta dos sons de cada criança. Os processos fonológicos foram organizados como tendo ou não a presença de cada processo.

Para este estudo, foram utilizados os relatórios do grau de severidade de fala, da análise contrastiva e dos processos fonológicos. Os resultados dos traços distintivos não foram apresentados, já que tais dados estão contemplados no relatório dos processos fonológicos, pois são o mesmo objeto analisados por perspectivas teóricas diferentes, e que resultam nos mesmos achados.

### Análise dos dados

Os resultados da etapa da análise da fidedignidade foram abordados de modo quantitativo. As análises foram realizadas no *software* SPSS, versão 28 para *Windows*.

#### Consistência interna

Para verificar a consistência interna foi realizado o cálculo do coeficiente *Alpha de Cronbach*, que é a medida mais utilizada para avaliar a confiabilidade^(18)^. Ela mede o grau de covariância entre os itens de uma escala e permite analisar a consistência do instrumento, calculando a correlação que existe entre cada item do teste e o restante dos itens ou o total dos itens^(17)^. Os valores variam de 0 a 1, sendo que valores superiores a 0,7 são considerados ideais, sugerindo uma adequada fidedignidade^(18)^. Já para valores superiores a 0,90, assume-se que há redundância ou duplicação, indicando que vários itens estão medindo o mesmo elemento do construto, sendo necessária uma eliminação desses itens redundantes^(21)^.

#### Confiabilidade interavaliadores

Para comparar a média da quantidade total de processos fonológicos e avaliar a conformidade dos juízes nos resultados da análise contrastiva e do grau de severidade de fala, foi executado o Coeficiente de Correlação Intraclasse (ICC). Ele é apropriado para medir a correlação das avaliações entre dois ou mais avaliadores quando há uma variável quantitativa. Os valores variam de 0 a 1. Quanto mais próximo de 1, maior a concordância entre os avaliadores. Se menor que 0,5, a concordância é fraca. Entre 0,5-0,75, é moderada. Entre 0,75-0,9, a concordância é boa. Se maior que 0,9, a concordância é excelente^(22)^.

#### Confiabilidade intra-avaliadores

Para avaliar a concordância dos juízes, ou seja, medir o grau de conformidade das avaliações entre dois momentos distintos, nos resultados do grau de severidade de fala e da análise contrastiva, foi utilizado o ICC. Os valores adotados como critério são os mesmos referidos da confiabilidade interavaliadores.

## RESULTADOS

As evidências de fidedignidade foram verificadas a partir dos resultados coletados pelo IAF. Para verificar a consistência interna do instrumento, os resultados foram analisados pelo coeficiente *Alpha de Cronbach* para cada fonema em cada posição silábica do Português Brasileiro (PB). O instrumento teve um coeficiente de 0,88, indicando alta consistência interna (acima de 0,7), evidenciando uma excelente fidedignidade dos escores que avaliam os fonemas do PB.

A Tabela 1 mostra a média da quantidade total de processos encontrados por cada avaliador, através do ICC. A concordância entre os avaliadores, quanto à quantidade de processos, teve médias variando entre 2,37 a 3,2 por criança, tendo amplitude de 0,65, e ICC apontando boa concordância, o que indica consonância entre os avaliadores em relação aos processos fonológicos encontrados nos participantes.

**Tabela 1:** Média da quantidade total de processos fonológicos entre os avaliadores

Avaliador						ICC	IC 95%
A		B		C			
M	DP	M	DP	M	DP		
2,50	1,99	3,02	2,06	2,37	1,85	0,89	0,84 0,92

Legenda: M = Média; DP = Desvio Padrão; ICC = Coeficiente de Correlação Intraclasse; IC = Intervalo de Confiança

Quanto às evidências de fidedignidade interavaliadores da análise contrastiva, a Tabela 2 exibe a confiabilidade entre os juízes, medida pela concordância da produção correta dos fonemas, em cada posição silábica do PB, através do ICC. A concordância teve valores acima de 0,90, para o /ʃ/ em OSI e OSM; o /ʒ/ em OSI e OSM; o /R/ em OSI; o /ʎ/ em OSM; o /r/ em OSM, OCI, OCM e CM; o /l/ em OCI e OCM; e o /s/ em CM. A concordância obteve valores entre 0,75 a 0,90, sendo considerada como boa, para a maioria dos outros fonemas, tal como demonstra a Figura 1.

**Tabela 2:** Concordância interavaliadores medida pela produção correta dos fonemas em cada posição silábica do PB por meio do ICC

**Fonemas**	**Posições**					
	**OSI**	**OSM**	**OCI**	**OCM**	**CM**	**CF**
**/p/**	0,42*	0,81	-	-	-	-
**/b/**	0,72	0,72	-	-	-	-
**/t/**	0,71	0,71	-	-	-	-
**/d/**	0,83	0,84	-	-	-	-
**/k/**	0,86	0,90	-	-	-	-
**/g/**	0,88	0,78	-	-	-	-
**/f/**	0,32*	-0,01*	-	-	-	-
**/v/**	0,73	0,63	-	-	-	-
**/s/**	0,71	0,89	-	-	0,91	0,47
**/z/**	0,71	0,86	-	-	-	-
**/ʃ/**	0,91	0,95	-	-	-	-
**/ʒ/**	0,93	0,92	-	-	-	-
**/m/**	-0,01*	0,36*	-	-	-	-
**/n/**	0,51*	0,82	-	-	0,76	0,41
**/ɲ/**	-	0,61	-	-	-	-
**/R/**	0,96	0,87	-	-	-	-
**/l/**	0,83	0,82	0,97	0,93	0,77	0,24*
**/ʎ/**	-	0,91	-	-	-	-
**/r/**	-	0,94	0,96	0,95	0,91	0,90

* Amostra é altamente homogênea

Legenda: OSI = Onset Simples Inicial; OSM = Onset Simples Medial; OCI = Onset Complexo Inicial; OCM = Onset Complexo Medial; CM = Coda Medial; CF = Coda Final

**Figura 1:** Relação da concordância interavaliadores acerca da produção correta dos fonemas em cada posição silábica do PB

Houve concordância moderada apenas em /b/ em OSI e OSM; /t/ em OSI e OSM; /v/ em OSI e OSM; /s/ em OSI; /z/ em OSI; /n/ em OSI; e /ɲ/ em OSM. Na maioria dos fonemas em que se obteve concordância considerada fraca, houve indicativo de amostra altamente homogênea.

A Tabela 3 apresenta a comparação interavaliadores e intra-avaliador referente ao resultado do grau de severidade de fala de cada criança, que foi mensurada pelo ICC. O resultado demonstrou que o ICC ficou acima de 0,90 em todas as ocorrências, indicando excelente concordância tanto entre os avaliadores, quanto entre cada avaliador em dois momentos distintos.

**Tabela 3:** Confiabilidade interavaliadores e intra-avaliador medida pelo grau de severidade de fala

Interavaliador	ICC	IC 95%
	0,97	0,96-0,98
**Intra-avaliador**	**ICC**	**IC 95%**
Avaliador A	0,98	0,95-0,99
Avaliador B	0,98	0,91-0,99
Avaliador C	0,98	0,94-0,99

Legenda: ICC = Coeficiente de Correlação Intraclasse; IC = Intervalo de Confiança

Em relação à fidedignidade intra-avaliador da análise contrastiva, a Tabela 4 expõe a confiabilidade de cada juiz, medida pela concordância da produção correta dos fonemas em cada posição silábica do PB, através do ICC. Foram elencados apenas os fonemas em que foi possível realizar a análise estatística, pois em alguns não havia variabilidade de desvio-padrão. O ICC só realiza a análise se houver tal variabilidade. Quando não há essa variabilidade, ou seja, concordância total, não é possível determinar o coeficiente estatístico por conta de características próprias da análise por ICC.

**Tabela 4:** Fidedignidade intra-avaliador medida pela produção correta dos fonemas em cada posição silábica do PB, através do ICC

Fonemas	Avaliador A	IC 95%	Avaliador B	IC 95%	Avaliador C	IC 95%
OSI /g/	0,92	0,80 - 0,97	0,69	0,16 - 0,88	0,96	0,89 - 0,98
OSI /v/	*	*	-0,25	-2,75 - 0,54	*	*
OSI /z/	0,89	0,72 - 0,99	0,76	0,37 - 0,91	0,37	-0,66 - 0,76
OSI /ʃ/	0,98	0,95 - 0,99	0,99	0,98 - 0,99	0,98	0,96 - 0,99
OSI /n/	*	*	0,36	-0,57 - 0,75	-0,15	-2,01 - 0,56
OSM /ʒ/	0,95	0,87 - 0,98	0,98	0,96 - 0,99	0,99	0,98 - 0,99
OSM /ɲ/	*	*	0,79	0,45 - 0,92	*	*
OSM /ʎ/	0,84	0,58 - 0,94	*	*	0,62	-0,00 - 0,85
OSM /r/	0,98	0,95 - 0,99	0,99	0,99 - 0,99	0,97	0,93 - 0,99
OCI /l/	0,98	0,97 - 0,99	0,99	0,98 - 0,99	0,98	0,95 - 0,99
OCI /r/	0,96	0,91 - 0,98	0,99	0,97 - 0,99	0,96	0,88 - 0,98
OCM /l/	0,97	0,92 - 0,99	0,99	0,97 - 0,99	0,96	0,91 - 0,98
OCM /r/	0,98	0,97 - 0,99	0,98	0,97 - 0,99	0,96	0,90 - 0,98
CM /s/	*	*	*	*	0,87	0,67 - 0,95
CM /l/	0,86	0,64 - 0,95	0,95	0,86 - 0,98	0,58	-0,14 - 0,84
CM /r/	0,80	0,48 - 0,92	0,98	0,96 - 0,99	0,81	0,49 - 0,93
CF /n/	*	*	0,76	0,40 - 0,91	*	*
CF /r/	0,92	0,76 - 0,97	0,93	0,83 - 0,97	0,86	0,63 - 0,94

* Não foi possível realizar o ICC por falta de variabilidade dos desvios-padrão

Legenda: OSI = Onset Simples Inicial; OSM = Onset Simples Medial; OCI = Onset Complexo Inicial; OCM = Onset Complexo Medial; CM = Coda Medial; CF = Coda Final; IC = Intervalo de Confiança

A concordância entre os avaliadores foi considerada excelente, com valores acima de 0,9 para a maioria dos fonemas. Houve vários fonemas com concordância classificada como boa, com valores entre 0,75 a 0,90, indicando uma adequada confiabilidade entre os juízes. O avaliador A teve concordância considerada como excelente ou boa em todos os fonemas elencados. O avaliador B teve o fonema /g/ em OSI considerado como moderado, com valores entre 0,50 a 0,75, além de ter tido os fonemas /v/ em OSI e /n/ em OSI classificados como fracos, com valores inferiores a 0,50. O avaliador C teve concordância considerada moderada para os fonemas /ʎ/ em OSM e o /l/ em CM, e concordância considerada fraca para o fonema /z/ em OSI.

## DISCUSSÃO

Há uma reconhecida relevância da utilização de instrumentos de avaliação de fala para a prática clínica fonoaudiológica^(23)^, porém, para que seja considerado confiável, é necessário passar por etapas de mensuração precisas, sendo elas: validade e fidedignidade^(15-19)^. Tendo em vista o objetivo deste estudo e seus resultados, o IAF mostrou-se como um pertinente e apropriado instrumento para analisar o sistema fonológico das crianças falantes de PB de forma detalhada, além de determinar o grau de severidade de TF, tendo indicadores adequados e satisfatórios de fidedignidade.

Em relação às evidências de fidedignidade do IAF, a consistência interna mostrou-se excelente, uma vez que ficou acima do indicador considerado minimamente ideal (0,70), indicando uma forte confiabilidade. Tal resultado evidencia ausência de redundância ou duplicação nos itens do instrumento. Outros instrumentos nacionais de avaliação fonoaudiológica também utilizaram o *Alpha de Cronbach* para analisar a consistência interna: o Instrumento de Avaliação Fonológica (INFONO)^(12)^, que faz a avaliação da fonologia; e a tarefa de discriminação de fala com pseudopalavras^(24)^, criada para avaliar a habilidade de discriminação auditiva de sons da fala. Ambos obtiveram resultados acima de 0,70, tendo indicadores considerados apropriados. Em um protocolo construído para avaliar a compreensão de linguagem oral de crianças de dois a seis anos^(25)^, a consistência interna variou de 0,60 e 0,70, classificada como moderada. Já para um estudo sobre a consistência interna da tarefa de avaliação de competências sintáticas^(26)^, o *Alpha de Cronbach* variou de 0,00 a 0,47, sendo insuficiente para validar a fidedignidade do instrumento.

Em um estudo de revisão sistemática^(27)^, que analisou os procedimentos de validação utilizados por instrumentos fonoaudiológicos de avaliação de linguagem oral, publicados em periódicos nacionais, ficou evidente que apenas cinco instrumentos, num total de 21, realizaram análises de fidedignidade pela consistência interna. Tais resultados demonstram a escassez de instrumentos validados em relação à confiabilidade, impedindo a exatidão de dados precisos na avaliação na prática clínica fonoaudiológica.

No estudo referido^(27)^, o cálculo amostral para definição do tamanho da amostra foi apresentado em apenas um estudo dos 21 incluídos na revisão sistemática, comprovando uma expressiva limitação nos estudos de validação de testes de fala. O IAF, por outro lado, demonstra tanto os indicadores de consistência interna, quanto o cálculo amostral, podendo ser considerado um instrumento confiável para a utilização na prática clínica e em pesquisas.

Os instrumentos necessitam passar por etapas de validade e fidedignidade para mensurar o que se propõe a medir e para que os resultados representem o construto analisado, para que não comprometa a precisão da avaliação e do diagnóstico^(12)^. Desta forma, um instrumento criado para avaliar o sistema fonológico precisa analisar todos os fonemas em todas as posições silábicas da sua língua alvo, para que se faça um correto julgamento sobre a presença ou ausência de diagnóstico de TF. Essa estrutura é preconizada no IAF para possibilitar uma análise minuciosa do perfil fonológico da criança, e pouco encontrado em outros testes de avaliação da linguagem. Seguindo esse mesmo parâmetro, foram encontrados apenas o Modelo Linguístico Fuzzy^(28)^, elaborado para classificar a gravidade do TF, e o já mencionado INFONO^(12)^.

Identificar os tipos de processos fonológicos e as substituições que a criança realiza é uma informação que colabora com a avaliação fonológica^(26,29)^, mas para que um instrumento possa adicionar essa análise, necessita ter a capacidade de avaliar todas as possibilidades fonêmicas em todas as posições silábicas de modo quali-quantitativo^(28)^. O IAF dispõe desse atributo, proporcionando informações detalhadas sobre os fonemas e todas as suas possibilidades de ocorrência, sendo um instrumento adequado para incluir a observação dos processos fonológicos.

Em relação às médias da concordância interavaliadores para processos fonológicos, os resultados indicaram uma fidedignidade adequada para essa análise. No entanto, cabe ressaltar que, apesar de ser um dado complementar para traçar a escolha do modelo de terapia, a análise dos processos fonológicos não é primordial, nem necessária^(2)^, uma vez que não explica, de forma detalhada e criteriosa, a organização do sistema fonológico e seu funcionamento com relação a cada fonema em cada posição silábica do PB, pois apenas dessa forma é factível e adequado delinear a conduta terapêutica^(29)^. Portanto, a análise do uso de processos fonológicos é um dado adicional para a avaliação, mas não fundamental, uma vez que a verificação da análise contrastiva fornece os aspectos norteadores e primordiais para saber o funcionamento do sistema fonológico de um indivíduo^(29)^.

Além da consistência interna, foi realizada a confiabilidade interavaliadores e intra-avaliador para testar a fidedignidade do instrumento. Um estudo internacional elaborado para avaliar a produção de fala em crianças turcas^(30)^ também seguiu as mesmas etapas para verificar as evidências de fidedignidade, assim como outra pesquisa nacional^(12)^.Em ambas as etapas, os indicadores do IAF apresentados no presente estudo para a análise do grau de severidade foram considerados excelentes, demonstrando que é um dado confiável para sua utilização nas avaliações e diagnósticos na prática clínica.

Também foram realizadas as duas etapas para a análise contrastiva do IAF, na qual se observou que a maioria dos fonemas apresentou confiabilidades entre excelentes e boas. No entanto, ambas as etapas tiveram alguns fonemas com indicadores moderados e fracos (Tabela 2). Em relação à confiabilidade interavaliadores, essa ocorrência pode ter sido afetada pelo indicativo de a amostra ser altamente homogênea, comprometendo a análise estatística e sendo difícil discriminar a produção dos fonemas entre os participantes da amostra. A homogeneidade da amostra pode afetar o ICC, e dessa forma, se a população da pesquisa for bastante homogênea, ou seja, quando são muito similares de acordo com uma determinada característica (resultando em um baixo desvio-padrão), há uma maior dificuldade para discriminar entre os indivíduos e o ICC se desvia de 1 (próximo de 0). Como exemplo, o fonema /p/ em OSI apresentou indicativo de amostra altamente homogênea, possivelmente por conta de falta de variabilidade das respostas dos sujeitos (dados deste fonema nesta posição foram praticamente idênticos para todas as crianças), e dessa forma, houve um baixo desvio-padrão, dificultando a realização do cálculo estatístico de ICC, desviando-se de 1. A variação encontrada na Tabela 2 é devido a variada amplitude de ICC encontrada entre os avaliadores.

Além disso, convém mencionar que a fidedignidade de um instrumento pode ser influenciada pela experiência e pelos critérios individuais de cada avaliador^(12)^. As discordâncias na confiabilidade interavaliadores da análise contrastiva podem ter ocorrido por conta da habilidade de oitiva e parâmetros próprios dos avaliadores. Isso pode ser verificado na confiabilidade intra-avaliador, uma vez que o avaliador B e C, mesmo tendo a maioria dos fonemas com fidedignidade excelente, tiveram alguns fonemas com indicadores moderados e fracos, enquanto o avaliador A teve apenas indicadores bons e excelentes (Tabela 4). Da mesma forma, outro estudo^(28)^ também teve indicadores variados entre os juízes, os quais afetaram os indicadores de concordâncias com vistas à confiabilidade. Porém, convém mencionar que os altos valores de ICC para a confiabilidade inter e intra-avaliadores do IAF na maioria dos fonemas apontam que há ótima concordância entre os juízes (Tabela 2). Assim, o IAF mostra-se confiável em classificar a fase de aquisição^(1)^ em que cada criança se encontra, em cada posição silábica, facilitando a avaliação e diagnóstico, como também o planejamento terapêutico e evolução individual.

Os instrumentos de avaliação da fonologia do PB mais aplicados e difundidos atualmente no país^(12)^ são o AFC^(13)^e o ABFW^(14)^, como já mencionado anteriormente, que são muito importantes como ferramentas para o processo diagnóstico dos fonoaudiólogos brasileiros. No entanto, apesar de tal importância, eles ainda não dispõem de estudos psicométricos de validade e de fidedignidade, o que poderia afetar a segurança de uso das medidas desses instrumentos. Desse modo, este estudo é pertinente para o avanço da fonoaudiologia, visto que o IAF torna-se uma opção válida e qualificada para se utilizar em avaliações de crianças com suspeita de TF e/ou com alterações no sistema fonológico, além de ser um instrumento que possibilita uma análise criteriosa e minuciosa de forma rápida, uma vez que o software produz rapidamente um relatório de desempenho.

O IAF foi elaborado visando contribuir com a prática clínica, auxiliando na avaliação da fonologia de crianças e apresentou ótimos resultados de fidedignidade. Apesar disso, este estudo possui algumas limitações, como: ser estruturado para falantes do PB em geral, mas os dados serem exclusivos de falantes de apenas uma amostra de uma escola pública do Rio Grande do Sul, podendo ser um obstáculo à generalização; não ter feito uso de índices padronizados para calibrar a avaliação de oitiva, permitindo critérios próprios dos avaliadores; e não apresentar coeficientes de fidedignidade por cada faixa etária, gênero e tipo de escola, uma vez que a variabilidade da amostra é composta apenas por estudantes oriundos do ensino público.

O presente estudo demonstrou que o IAF é um instrumento confiável para a utilização e aplicabilidade, trazendo evidências de fidedignidade dos escores, porém, outros estudos deverão ser realizados em busca de outras evidências de validade, como a etapa de consequências do teste, a normatização, a padronização e a análise de itens. Cabe ressaltar que o *software* do IAF foi desenvolvido para avaliar qualquer faixa etária, porém, ele foi validado para crianças com idade superior a cinco anos, respeitando o período de estabilização da aquisição fonológica e o conjunto de sinais que caracterizam o TF, além da idade da amostra.

## CONCLUSÃO

O IAF apresentou evidências robustas de fidedignidade, demonstrando bons escores de consistência interna, o que aponta uma excelente confiabilidade dos itens do instrumento, além da confiabilidade inter e intra-avaliador. Desta forma, houve uma adequada concordância entre os resultados fornecidos pelo IAF, tanto em aplicação por diferentes avaliadores, quanto por aplicação individual em momentos distintos. Por ser um estudo que seguiu parâmetros psicométricos de validação, as evidências apresentadas demonstram a qualidade do instrumento e fortalecem a segurança do uso de suas medidas. Conclui-se que o IAF é uma opção confiável e segura para ser utilizado em pesquisas brasileiras e na prática clínica para a avaliação do sistema fonológico de crianças.

## Footnotes

a Pesquisa sendo desenvolvida por Ana Carolina Sartori Bernardi, em 2022, na Universidade de Ciências da Saúde de Porto Alegre.

b Estudos psicométricos sobre as propriedades do instrumento de validade de conteúdo, processo-resposta e validade de construto estão em produção.
